# Inflammatory Mediators in the Oral Fluids and Blood Samples of Type 1 Diabetic Patients, According to Periodontal Status—A Systematic Review

**DOI:** 10.3390/ijms26062552

**Published:** 2025-03-12

**Authors:** Álvaro Parra Meder, Rosana Costa, Paula López-Jarana, Blanca Ríos-Carrasco, Marta Relvas, Filomena Salazar

**Affiliations:** 1 School of Dentistry, Universidad de Sevilla, C/Avicena s/n, 41009 Sevilla, Spain; alvparmed@alum.us.es; 2 Department of Medicine and Oral Surgery, University Institute of Health Sciences (IUCS-CESPU), 4585-116 Gandra, Portugal; rosana.costa@iucs.cespu.pt (R.C.); paula.jarana@iucs.cespu.pt (P.L.-J.); marta.relvas@iucs.cespu.pt (M.R.); filomena.salzar@iucs.cespu.pt (F.S.); 3 Oral Pathology and Rehabilitation Research Unit (UNIPRO), University Institute of Health Sciences (IUCS-CESPU), 4585-116 Gandra, Portugal

**Keywords:** immunology, type 1 diabetes, periodontal diseases, inflammatory mediators, cytokines, immune response

## Abstract

There is currently little information on the immune profile of adult type 1 diabetes patients diagnosed with periodontal disease. The aim of this systematic review is to bring together the known evidence of which inflammatory markers, measured in salivary flow or gingival crevicular fluid and serum blood, are present in both pathologies. Following the Preferred Reporting Items for Systematic reviews and Meta-Analys guidelines, we systematically searched in the PubMed, Web of Science, Scopus and Cochrane Library databases for studies on the associations of different chemokines with type 1 diabetes mellitus and periodontal disease. From a total of 703 patients, of which 526 were patients diagnosed with type 1 diabetes and 215 were controls without diabetes, multiple inflammatory mediators, such as interleukin 8, which showed higher concentrations in the crevicular fluid in several studies of type 1 diabetes patients and a greater severity in its effects on the periodontal status, as well as osteoprotegerin and tumor necrosis factor alpha, have been found elevated in diabetic patients with poor periodontal control. The results suggest that interleukin 8, tumor necrosis factor alpha and osteoprotegerin may be promising novel biomarkers of type 1 diabetes mellitus, and may also indicate the susceptibility profile in these individuals for the worsening of the patient’s periodontal status.

## 1. Introduction

Type 1 diabetes mellitus (T1DM), as a chronic autoimmune disease, is characterized by the selective loss of insulin-producing beta cells (β cells) mediated by T cells [1,2]. The progressive destruction of these pancreatic beta cells mediated by an altered immune response is characteristic of T1DM. This destruction leads to a deficiency in insulin production resulting in hyperglycemia and hyperlipidemia. However, its etiology is still unclear; it seems to be the result of a combination of genetic and environmental factors, including some viruses. The nature of autoimmunity involves the dysfunction of the innate and acquired responses [3,4,5,6].

The role of the inflammatory effect of certain proinflammatory cytokines concentrated in pancreatic beta cells supports the thesis of their contribution to the development of type 1 diabetes [5]. It is still under debate what the sequence is and what the implications of the genetic side are that could induce cell destruction [7,8].

There seem to be two types of type 1 diabetes: autoimmune (where there is a severe deficit of insulin secretion due to the damage of insulin-producing beta cells in the pancreas) and idiopathic (where no autoimmune mechanisms are involved, and the cause is currently unknown) [8,9].

Periodontal disease is a pathology of multifactorial etiology where certain periodontopathogenic bacteria alter the balance of the microbiome and infect the tissues in such a way as to trigger an inflammatory response [10]. Depending on the inflammatory response triggered in the individual, it can remain as only an acute inflammation limited to the soft tissues (gingivitis) or become chronic and include the destruction of the supporting tissues [11,12,13]. The hyperglycemic state and hyperlipidemia in turn activate glycation end products (AGEs), whose receptors are on monocytes, endothelial cells and macrophages, which secrete interleukin 1β (IL-1β), interleukin 6 (IL-6), Prostaglandin E2 (PGE2) and tumor necrosis factor alpha (TNF-α) [14,15].

Meanwhile, several studies with diabetic patients have shed some light on the role of cytokines and other inflammatory mediators in the pathogenesis of periodontitis [16,17,18,19]. Early diagnosis of periodontal disease in patients with T1DM is a crucial key to improve the patient’s systemic health, limiting the damaged caused by the hyperglycemic state [8,20,21,22].

Clinical studies have provided evidence that higher levels of proinflammatory mediators within the gingival tissues of diabetic patients, like IL-1β, TNF-α, IL-6, the receptor activator of nuclear factor-kappa B ligand (RANK-L)/osteoprotegerin (OPG) ratio and oxidative stress play a significant role in the increased periodontal destruction [16]. This was also supported by studies using animal models or cell cultures exposed to high glucose levels [23].

Linhartova et al. found that interleukin 8 (IL-8), a C-X-C motif (CXC) member of the cytokine family, is one of the major chemokines and activators of neutrophils with the interaction of CXCR1 (C-X-C motif chemokine receptor 1) and CXCR2 (C-X-C motif chemokine receptor 2) [24]. IL-8 is related to the initiation and amplification of acute inflammatory reactions and is secreted by various types of cells. Chemokines and neutrophils have been previously associated or implicated with the pathogenesis of type 1 diabetes. However, it is not clear what the association itself is [20]. The levels of IL-8 in oral keratinocytes, and cells of the gingival epithelium, crevicular fluid, plasma and serum have been ambiguous in patients with T1DM and periodontitis; authors such as Linhartova found an association between IL-8 and patients with periodontitis [24,25].

Salivary biomarkers have been used for the early diagnosis of several diseases, including diabetes, specifically immunoglobulin A (IG-A) [26,27]. In addition, other cytokines such as IL-6 and TNF-α [8,28] have also been associated with an immune response influenced by chronic inflammation, which represents a complication in diabetic patients. Specifically, immunoglobulin 6 (IG-6) and TNF-α are potent proinflammatory cytokines that are significantly increased in patients with gingivitis and type 1 diabetes. At the plasma level, other mediators have been studied, such as IL-8, which some authors have found in higher concentrations in patients with type 1 diabetes [24].

The mechanisms by which diabetes affects periodontitis are not fully understood; however, it is known that diabetes is correlated with an improvement in the progression of periodontitis in part due to the increased inflammatory response. A deeper understanding of the two-way relationship between periodontal diseases (PD) and diabetes will contribute decisively to designing new treatment strategies and guiding future lines of research. For that reason, the main objective of our study was to review the current evidence of the role of cytokines, chemokines or immune biomarkers in the bidirectional relationship between type 1 diabetes and periodontal disease.

## 2. Results

**Figure 1:**
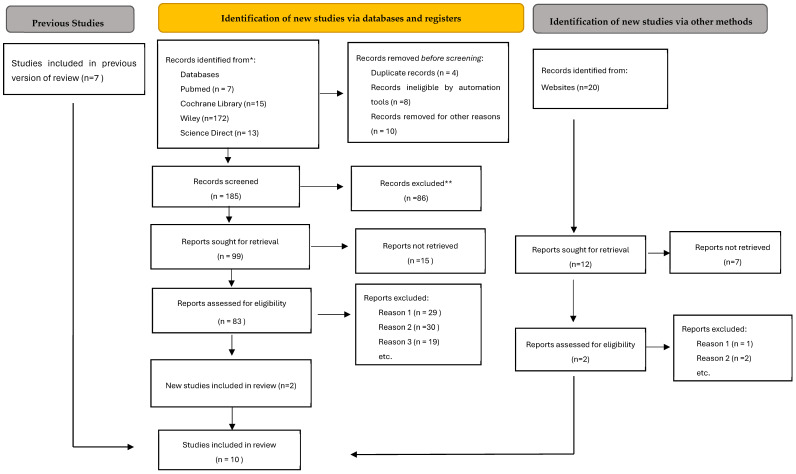
Flow diagram of study selection. **Reason 1:** in vitro studies. **Reason 2:** not specifying whether and when any previous periodontal treatment was performed on the patient in a way that could alter the results obtained. **Reason 3:** the population included patients with type 2 diabetes. * Consider, if feasible to do so, reporting the number of records identified from each database or register searched (rather than the total number across all databases/registers). ** If automation tools were used, indicate how many records were excluded by a human and how many were excluded by automation tools.

From a total of 207 articles, after eliminating duplicates and articles whose title and abstract did not fit our inclusion criteria, a total of 10 articles were chosen for review. The flow chart is described in Figure 1 according to the criteria of Page et al. [29]. The total number of studies were eight cross-sectional studies (Table 1) and two cohort studies (Table 2), and specifically, one of which had a 50-year follow-up (Table 3).

## 3. Discussion

Type 1 diabetes mellitus is an inflammatory disease with autoimmune characteristics that is experiencing an exponential increase in its prevalence worldwide [13]. Periodontal disease is the sixth most common systemic complication associated with type 1 diabetic patients [16]. A study carried out in 2019 by Shinjo et al. [36] on a cohort of 170 patients with a diagnosis of T1DM who were over 50 years old and belonged to the Joslin Medalist group aimed to discover the protective factors that this population presented in terms of ocular, renal and neurological complications, compared to the rest of the diabetic patients in the USA. This study found that the prevalence of severe periodontal disease was 13.5%, significantly lower than the overall rate of 23.3% in the US for patients aged 65–74 years in the NHANES (2009–2012). However, this could also be related to the narrow range of HbA1c exhibited by the Medalists, with very good glycemic control (mean 7.15%) compared to the national mean of HbA1c in T1DM (8.2% overall and 7.6% in those ≥50 years age) [36].

Current investigations have proposed that one of the biological mechanisms that occurs in diabetic patients with a chronic hyperglycemic state results in the formation of advanced glycation end-products (AGE) through the irreversible non-encapsulated formation of proteins and lipids. This results in the loss of functionality of proteins, leading to molecules that stimulate the inflammatory mediators through the interaction with AGE receptors (RAGE).

On the one hand, the receptors of triggers of AGE receptors such as monocytes, endothelial cells and macrophages are associated with immune and non-immune T1DM. These immune cells produce a significant increase in cytokines and the proinflammatory mediators interleukin IL-1β, IL-6, PGE-2 and TNF-α, which causes the destruction of periodontal tissues, because in the periodontium, there are numerous cells which express RAGE, like oral keratinocytes, fibroblasts, macrophages and monocytes. This interaction between AGE and AGE receptors will stimulate matrix metalloproteinases (MMPs) and other osteolytic factors that will cause damage to the periodontal ligament and periodontal bone matrix [30,37]. On the other hand, hyperlipidemia also increases the amount of IL-1β and TNF-α [30].

The increase in IL-6 and TNF-α in synchrony promotes the increase in C-reactive proteins and the decrease in adiponectin, which will increase the insulin resistance characteristic of a diabetic patient. In fact, TNF-α amniotic acid together with increased IL-1β results in increased protein kinase C (PKC), the destruction of pancreatic β-cells and a synergistic increase in hyperlipidemia through lipid clearance, increased lipogenesis and decreased lipolysis.

The study by Shinjo et al. showed that serum IL-6, but not C-reactive protein (CRP) levels, is associated with an increased periodontitis severity in this group of periodontitis patients [36]. However, other researchers found an increased trend of CRP and HbA1c in type 1 adults, which reflects data from individuals with type 2 diabetes and type 1 pediatric patients (HbA1c data only) [1].

Specifically, according to Lappin et al., IL-6 is a pleiotropic proinflammatory cytokine found in elevated circulating levels in poorly controlled T1DM patients and also appears to be related to periodontitis [37].

IL-8 is a major neutrophil chemoattractant and plays a key role in the induction and maintenance of inflammation. High levels of these cytokines have been detected in the gingival tissues of patients with periodontal disease and T1DM [24].

In relation to serum OPG, it is still not clear if it increases in cases of attachment loss and greater severity. Other studies conclude that the local concentration of RANK-L/OPG increases in healthy patients, patients with gingivitis and periodontitis. There are studies that state that the samples of control patients with the same periodontal situation are small and therefore no conclusions can be drawn about the effect of serum OPG in relation to the status and degree of PD [31].

Studies carried out in animal models have demonstrated higher levels of interleukin 23 (IL-23) and interleukin 17 (IL-17) in patients with type 1 diabetes, and in addition, the interferon (IFN) produced by T cells appears to be implicated in the destruction of Langerhans cell islets. In the same way, IL-17 is found in higher concentrations in patients with periodontitis [11,38]. Salivary biomarkers have been verified as a non-invasive diagnostic strategy. Our other preliminary analysis of saliva samples recovered from PD patients with distinct degrees of severity was performed to select the most informative panel of inflammatory mediators. We observed a significant increase in IL-1β,, IL-6 and interferon gamma (IFNγ) in the saliva of PD patients compared to age- and gender-matched healthy controls. In opposition, we observed the inverse phenotype with interleukin-1 receptor antagonist (IL-1Ra) and interferon gamma-induced protein 10 (IP-10) [39].

Among the plethora of markers, MMP-8 is also known as collagenase 2 or neutrophil collagenase and members of the MMP family have been studied extensively in oral fluids. MMP-8 is the main collagenase and is found in the inflamed gingiva of adults, being associated with the pathological destruction of the extracellular matrix. A recent review of 61 articles stated that MMP8 has the potential (alone or in combination with other inflammatory and microbiological markers) to serve as a diagnostic tool in periodontal and peri-implant disease [30,31,32].

Chronic hyperglycemia is related to the increased production of AGEs. These products compromise physiological and mechanical function resulting in a malfunctioning ECM (extracellular matrix), e.g., they suppress collagen production from periodontal and gingival ligament fibroblasts. In an in vivo study, they have also been shown to be increased in type 1 diabetes (AGE+). The hypothesis says that in diabetic patients, there are a greater number of local inflammatory biomarkers than in non-diabetic patients. This study evaluates the levels of MMP8, interleukin 8 (IL-8) and AGEs in GCF in type 1 diabetes with different glycemic levels compared to healthy controls [30]. Interleukin 8 has been identified as a chemotactic factor, produced by various cell types upon the stimulation and induction of neutrophils that release lysosomal enzymes. IL-8 levels in crevicular fluid have been found to be significantly higher in patients with chronic PD but decreased in those treated periodontally [30]. This is in line with other studies on crevicular fluid, where an increase was also found in patients with periodontitis with a higher degree of severity [37].

Duque et al. reported higher lipid parameters in type 1 diabetes patients compared to controls; however, they obtained similar results in red complexes, IL-1β, TNF-α and IL-6. On the other hand, another author who used protein analysis reported on eight proteins [33,35].

The immune system’s neutrophils, macrophages and monocytes are among the cells whose activities are compromised by diabetes mellitus (DM).

Gingival crevicular fluid changes in connection with immune cell activity, and modifications in the control of proteolytic production and modified inflammatory cytokine profiles have all been found in smokers’ studies. Smoking affects the immune system (delaying the migration and recruitment of neutrophils), the microbiota (the composition of subgingival biofilms varies with the increased prevalence of periodontal pathogens) and the healing capacity of the periodontium (higher collagenolytic activity coupled with fewer gingival blood vessels) [40].

Tobacco use is associated with a number of diseases and increases the risk of developing periodontitis. It has been shown that smokers suffer more periodontal disorders than non-smokers, and the impact of tobacco on periodontal tissues has been debated for decades [34,35,36,37,38,39,40].

It would be necessary to study a greater number of inflammatory biomarkers in different media, both minimally invasive, such as crevicular fluid or saliva, as well as more invasive media, such as blood samples. A correct periodontal evaluation should be carried out in these patients with standardized diagnostic criteria in order to compare different populations. The risk of taking records of deep pockets has been criticized in several studies, and healthy sites are therefore also necessary to compare results. The importance of these studies in the public health of the population is great, since in addition to periodontal involvement, a higher prevalence of neuropathies related to a greater increase in IgA or cardiovascular risk systematically associated with IL-6 and CPR have been recorded [36].

## 4. Materials and Methods

The study protocol for this systematic review was registered on the International Prospective of Systematic Reviews (PROSPERO), under number CRD42024565892.

This systematic review was conducted from 2010 to 2024, according to the “Preferred Reporting Items for Systematic Reviews and Meta-Analysis guidelines” (PRISMA) [41] using the databases MEDLINE via PubMed and Cochrane Library, Web of Science, and Science Direct. The search was also conducted using the following journals: Journal of Clinical Periodontology, Journal of Periodontology and Periodontology 2000 via Wiley Online Library (2012 to present). The search strategy was as follows:

(((chemotactic cytokines[MeSH Terms]) OR (inflammation mediators[MeSH Terms]) OR (interleukins[MeSH Terms])) AND (periodontal disease[MeSH Terms]) AND (type 1 diabetes mellitus[MeSH Terms])).

To demonstrate intra- and inter-examiner reliability, two independent examiners (A.M./F.S.) were used and the Kappa coefficient test produced nearly perfect agreement (0.81–0.99) during the search and selection of the articles. Disagreements between these examiners were later resolved by agreement and based on the opinion of a third experienced researcher (M.R.)

PICO question: What inflammatory markers have been identified in adult patients with type 1 diabetes and periodontal disease?

The records were screened by the title, abstract and full text by two independent investigators. The studies included in this review matched all the predefined criteria according to PICOS at Table 4 (“Population”, “Intervention”, “Comparison”, “Outcomes” and “Study design”). A detailed search flow chart is presented in the Results section.

The eligibility criteria were organized, using the PICO method, as follows:

The inclusion criteria corresponding to the PICO questions were articles in English, Portuguese or Spanish; articles related to T1DM; and cross-sectional studies, case–control studies, cohort studies and randomized controlled clinical studies. The studies chosen were from 2010 to 2024 to rule out outdated study methodologies or diagnostic tests.

On the other hand, the exclusion criteria were articles without an abstract available; literature reviews and meta-analyses, expert opinions, letters to the editor, conference abstracts and animal studies; and studies investigating type 2 diabetes mellitus (T2DM) exclusively. We also excluded inflammatory diseases, chronic liver disease or articles related to any treatment that may modify the study parameters, such as antibiotics, immunosuppressants or antiepileptic drugs. The pre-selected studies were excluded for several reasons, including the population being children or adolescents; the population being patients with type 2 diabetes and not specifying this in the title; and not specifying whether and when any previous periodontal treatment was performed on the patient in a way that could alter the results obtained.

### 4.1. Extraction of Sample Data

The data were collected by drawing up a results table, and the information was collected, taking into consideration the study design and aim, the eligibility criteria, the study population (with sample size and age group or average age), the duration in months or years of the study as well as the follow-up period, the outcome measures and the results.

### 4.2. Study Quality and Risk of Bias

To assess the methodological quality of a study and to determine the extent to which a study had addressed the possibility of bias in its design, conduct, or analysis, we used the Joanna Briggs Institute (JBI) guidance 2017 for each type of study (cross-sectional, case–control, cohort studies or randomized controlled trials) [42]. For each type of study, a different questionnaire was conducted using the answers Yes (Y), No (n), Unclear (UN) and Not Applicable (NA). Two independent examiners (A.M./P.J.) were used to demonstrate intra- and inter-examiner reliability. All cross-sectional studies responded positively to most of the bias test responses; only two questions were not answered positively, having a low risk of bias. The two cohort studies responded positively to all responses (Table 1 and Table 2).

## 5. Conclusions

Since type 1 diabetes is a disease with an etiology highly induced by the immune response, we believe that there is a deficit in knowledge specifically in adults, a field that has received little study, and not in children or adolescents. If we also add the fact of its association with periodontal disease, we will see that there are very few studies that evaluate the profile of inflammatory cytokines in these individuals.

The results suggest that IL-8, TNF-α and OPG may be promising novel biomarkers of T1DM and may also indicate the susceptibility profile in these individuals for the worsening of the patient’s periodontal status. Future research should attempt to replicate these findings in longitudinal studies and explore the potential mechanisms underlying other biomarkers as IL-1β, IL-6, IL-23, IFNγ, IL-1RA and IP-10, as well as IL-10, IL-17, IL-33, RANK- L and their associations.

## Figures and Tables

**Table 1:** Joanna Briggs Institute Critical Appraisal Checklist for Analytical Cross-Sectional Studies.

	1.	2.	3.	4.	5.	6.	7.	8.
Sereti et al., 2020 [30].	Y	Y	Y	Y	N	N	Y	Y
Linhartova et al., 2018 [24].	Y	Y	Y	Y	N	N	Y	Y
Steigmann L. et al., 2022 [1].	Y	Y	Y	Y	Y	Y	Y	Y
Antonoglou et al., 2013 [31].	Y	Y	Y	Y	Y	Y	Y	Y
Popławska-Kita A. et al., 2014 [32].	Y	Y	Y	Y	Y	Y	Y	Y
Duque et al., 2017 [33].	Y	Y	Y	Y	N	N	Y	Y
Maksymenko et al., 2021 [34].	Y	Y	N	Y	Y	Y	Y	Y
Keles et al., 2020 [35].	Y	Y	Y	Y	Y	Y	Y	Y

**Table 2:** Joanna Briggs Institute Critical Appraisal Checklist for Cohort Studies.

	1.	2.	3.	4.	5.	6.	7	8.	9.	10.	11.
Shinjo et al., 2019 [36].	N	N	Y	Y	Y	Y	Y	Y	Y	Y	Y
Lappin et al., 2015 [37].	Y	Y	Y	Y	Y	Y	Y	Y	Y	Y	Y

**Table 3:** Main results for the studies included.

Author	Study Design	Study Aim	Inclusion Criteria	Exclusion Criteria	Sample Size	Age Group	Study Duration	Outcomes Measured	Results
Sereti et al., 2020 [30].	Cross-sectional study	To compare the levels of GCF, IL-8, matrix metalloproteinase 8 (MMP-8) and AGEs in a cohort of T1DM subjects and healthy controls.	Subjects were aged between 18 and 85 years old, presented at least 10 natural teeth and were diagnosed with T1DM for more than 1 year.	(NR)	Fifty subjects with a diagnosis of T1DM were recruited from the patient cohort. Subjects aged between 18 and 85 years old, presented at least 10 natural teeth and were diagnosed with T1DM for more than 1 year. Control group of 50 systemically healthy subjects matched for age, sex and smoking status.	18–65 years	Transversal study	GCF, IL-8, MMP-8 and AGEs, and periodontal parameters PI, GI, PD, BOP, REC, furcation and tooth mobility.	The GCF levels of IL-8, MMP-8 and AGEs did not differ significantly between the groups. Further analysis of the GCF markers in younger (<40 years) and older (≥40 years) cohorts revealed no significant differences between younger diabetics and controls or between older diabetics and controls.
Steigmann L. et al., 2022 [1].	Cross-sectional study	To identify salivary biomarkers that are associated with PD and measures of diabetic autonomic dysfunction.	Age-matched healthy non-obese controls with normal glucose tolerance, normal blood pressure and cholesterol.	Presence of any retinopathy, nephropathy, peripheral and autonomic neuropathy, and cardiovascular disease.	H 10 DM 10 DMN 12	(40 ± 12, 44 ± 16 and 56 ± 11 years old in H, DM and DMN, respectively)	(NR)	IgA	The mean salivary IgA level inT1 DM was 9211.5 ± 4776.4 pg/mL, which was significantly lower than HC (17,182.2 ± 8899.3 pg/mL). IgA in DPN patients with a healthy periodontium was significantly lower (5905.5 ± 3124.8 pg/mL) compared to HC, although IgA levels in DPN patients with gingivitis (16,894.6 ± 7084.3) were not. IgA and periodontal condition were the indicators of the binary response given by HC versus T1DM, and HC versus DPN, respectively.
Antonoglou et al., 2013 [31].	Cross-sectional study	To explore in a cross-sectional study design whether any association exists between the extent of periodontal tissue destruction and circulating levels of sRANKL and OPG.	T1DM adult patients.	Previous antibiotic treatment (4 months). Immunosuppression patients who need antibiotic prophylactic.	80 patients with T1DM and 56 nonsmokers. The duration of insulin therapy was 20.0 years (range 1.2– 48.0 years, median 18.3 years).	38.6 ± 12.3 years	NR	CAL, bleeding and PD 4 mm or mean pocket depth were not significantly related to the serum levels of sRANKL and OPG and the sRANKL/OPG ratio.	A potential confident association between serum OPG and periodontal attachment loss/severity of periodontal disease in subjects with T1DM was confirmed.
Shinjo et al., 2019 [33].	Cohort study	To explore a subset of the Medalist cohort, who have been shown to have protection from other diabetic complications, to assess whether they are also protected from the development of periodontitis, a known complication of diabetes.	Joslin Medalist group with more than 50 years of diagnosed T1DM.	NR	170 patients from a 50-year Medalist study were examined between 2008 and 2010.	Mean age of 64.6 ± 6.9, age at diagnosis of 11.0 ± 6.5 years and duration of T1DM of 53.4 ± 4.3 years.	From 2008 to 2015 or death.	PI GI (REC) PD CAL BOP IG *P. gingivalis*	Detectable serum C-peptide was associated with higher PPD and CAL. Serum IL-6 levels and Ig titers against *P. gingivalis* found a positive association.
Lappin et al., 2015 [34].	Cross-sectional study	To compare the circulating levels of IL-6, IL-8 and CXCL5 in patients with T1DM, with and without periodontitis, to control groups consisting of systemically healthy, non-smoking individuals with and without periodontitis.	Minimum two sites with PPD and CAL > 5 mm and without periodontitis treatment.	No history of smoking within past 5 years. Pregnancy. Immunosuppression. Taking antibiotics or anti-inflammatory medication for 6 weeks. Less than 20 teeth.	104 participants in the study; 19 healthy volunteers, 23 patients with periodontitis, 28 patients with T1DM and 34 patients with T1DM and periodontitis.	Aged 20–56 (mean 36.4 ± 9.9)	2007–2010	AGE end product production.	Patients with diabetes and periodontitis had higher plasma levels of IL-8 than patients with periodontitis alone. Plasma levels of IL-8 correlated significantly with IFCC units, clinical probing depth and attachment loss. AGE and LPS, alone or in combination, stimulated IL-6, IL-8 and CXCL5 expression in both OKF6/TERT-2 cells and THP-1 monocytes.
Linhartova et al., 2018 [24].	Cross-sectional study	To determine IL-8 in plasma, CXC R2 polymorphisms and the presence of T1DM selective bacteria.	Willingness to participate, and compliance criteria of T1DM and CP, without periodontal treatment.	Coronary artery diseases, malignancies, immunodeficiency disorders, pregnancy or lactation, immunosuppression drugs, <20 teeth and the inability to consent.	153 patients (73 healthy, 80 diabetics)	The mean ages and BMI were similar for patients with T1DM + CP and HC, but there were significant differences in mean ages between groups of HC + CP/T2DM + CP and non-periodontitis HC (mean standard deviation, SD: 59.5 ± 9.3/66.8 ± 8.5 vs. 45.5 ± 9.6, *p* < 0.01).	NR	Sample collection and plasma level analysis (IL-8). Genetic analysis: genomic DNA. Periodontal bacteria analysis: red complex.	Mean BMI was significantly higher in T2DM + CP patients than in non-periodontitis HC and T1DM + CP patients (mean ± SD) (29.9 ± 7.7 vs. 23.8 ± 4.2/25.1 ± 3.1, *p* < 0.05). Smoking status did not differ between T2DM+CP and non-periodontitis HC (5.3% vs. 7.1% smokers) or between T1DM + CP patients and HC + CP individuals (19.4% vs. 25.0% smokers). DR was present only in patients with T1DM + CP, and DPN was also present significantly more frequently in this group of patients (*p* < 0.01). Diabetic patients with CP had significantly higher IL-8 than HC + CP (11.02 pg/mL (6.47–15.17), *p* < 0.05. IL-8 and CXC R2 had no association.
Popławska-Kita et al., 2014 [32].	Cross-sectional study	They assessed oral hygiene and periodontal status in healthy individuals and patients with T1DM in relation to (i) their glycemic control, (ii) smoking and (iii) serum inflammatory biomarkers.	All patients and controls gave their informed consent to participate in the study and the protocol was approved by the local ethics committee.	Presence of systemic diseases other than type 1 diabetes that could influence the progression of PD, along with the intake of immunosuppressive drugs, steroids or non-steroidal anti-inflammatory drugs, pregnancy and fixed orthodontic appliances.	107 patients for T1DM	T1DM good metabolic control HbA1c < 6.5% (34.8 ± 10.9). T1DM poor metabolic control HbA1c > 6.5% (37.9 ± 3.7.) Control group (32.3 ± 1.0).	NR.	Age, weight, systolic and diastolic pressure, Hb A1c, fasting glucose, total cholesterol, HDL, LDL, triglycerides, CRP, IL-1, TNF-α, number of teeth, OHI, CPI.	In the patients with T1DM, OHI correlated with blood pressure and serum TNF-α level, and negatively with number of teeth.
Duque et al., 2017 [33].	Cross-sectional study	To compare the prevalence of periodontal pathogens, systemic inflammatory mediators and lipid profiles in T1DM children with NDM children, both with gingivitis.	Children recruited from the Pediatric Dentistry Clinic.	Patients undergoing antibiotic prophylaxis for dental treatment, uncontrolled systemic diseases, immunological compromise, subjects who were wearing orthodontic devices, subjects who had been undergoing periodontal treatment 12 months before the beginning of the study, those who had been taking antibiotics within 6 months prior to the clinical examination, those with extensive caries lesions, individuals who were using an antiseptic solution in the past 3 month period and smokers.	27 DM children and 24 NDM children	7–13 years	NR	PPD GI PI Il-1β TNF-α IL-6	There were no statistically significant differences in the periodontal indices of patients with DM and NDM, indicating similar periodontal status. In both groups, similar levels of TNF-α, IL-6 and Il-1β in GCF were found.
Maksymenko et al., 2021 [34].	Cross-sectional study	To study the levels of proinflammatory IL-18 in the oral fluid of T1DM children, and to determine their periodontal status and the level of oral hygiene.	Children with or without T1DM	Children receiving or who had previously received orthodontic treatment; smokers; periodontal or antibiotic medication during the previous six months; any other systemic disorders other than diabetes mellitus; and eruptive gingivitis. Children with diabetes mellitus who had any problems other than periodontal inflammation were also not included in our study.	82 children: 56 children with T1DM and 26 NDM. Group 1–13 contained children with clinically healthy periodontium and no concomitant diseases; Group 2–13 contained children without concomitant diseases, but with chronic catarrhal gingivitis; Group 3–26 contained children with T1DM and healthy gums; and Group 4–30 contained children with T1DM and chronic catarrhal gingivitis.	6–12 years old	NR	OHI GI BOP IL-18	Children with chronic gingivitis and T1DM showed the highest level of IL-18 in oral fluid (70.91 ± 7.48 pg/mL). Increased values of IL-18 in oral fluid were associated with the presence of T1DM in children.
Keles et al., 2020 [35].	Cross-sectional study	To evaluate the levels of IL-18 and TNF-α in the GCF of T1DM children with gingivitis.	Children aged between 8 and 14 years; diagnosed with T1DM with an HbA1c level < 7.5% at least 12 months prior to the study, by a pedi- atric endocrinologist; exhibiting their first molars and maxillary and mandibular incisors that are fully erupted, free of cavities; and those not having any systemic disorders (for the healthy group).	Children with any other identified systemic chronic diseases; with an HbA1c level greater than 7.5%; requiring restorative and endodontic therapy; taking immunosuppressive medications within the last six months; taking any medication on a regular basis; using orthodontic appliances; having clinical attachment loss; and having any destructive periodontal disease or periodontal therapy involving antimicrobial or anti-inflammatory drugs.	88 C=children: 44 T1DM, 44 systemically healthy. (1) Systemically and periodontally healthy children (H, n = 22), (2) systemically healthy children with gingivitis (G) (n = 22), (3) periodontally healthy children with T1DM (T1DM + H, n = 22) and (4) children with T1DM and gingi- vitis (T1DM + G, n = 22).	8–12 years old	April–June 2019	PI GI PPD GCF TNF-α	Diabetic children exhibited similar amounts of TNF-α and GCF IL-18 in comparison with systemically healthy children (*p* > 0.05). Total GI, PI, PPD, GCF volume and TNF-α levels were substantially higher in the gingival subgroups than in the H subgroups (*p* < 0.0001). Compared to the periodontally healthy subgroups, the gingivitis subgroups had noticeably lower IL-18 concentrations. A strong relationship between gingival inflammation and TNF-α was confirmed by the presence of high levels of TNF-α in the GCF in children with gingivitis.

Legend: AGEs—advanced glycation end-products; BOP—bleeding on probing; BMI—mean body mass index; CAL—clinical attachment Loss; CP—chronic periodontitis; CPI—Community Periodontal Index; CRP—C-reactive protein; CXCL5—C-X-C motif chemokine 5; CXCR2—C-X-C motif receptor 2; DM—diabetes mellitus; DNA—deoxyribonucleic acid; DPN—neuropathy; DR—retinopathy; GCF—gingival crevicular fluid; GI—Gingival Index; HbA1c—glycated hemoglobin; HC—healthy controls; HDL—high density lipoprotein; IFCC—levels of blood glucose/glycated hemoglobin; IgA—immunoglobulin A; IL-1—interleukin 1; IL-6—interleukin 6; IL-8—interleukin 8; IL-18—interleukin 18; LDL—low density lipoprotein; LPS—lipopolysaccharide; MMP-8—matrix metalloproteinase 8; NR—non-referred; NDM—no diabetes mellitus; OHI—Oral Hygiene Index; OKF6/TERT-2—immortalized human oral keratinocyte cell line; OPG—osteoprotegerin; PD—periodontal disease; PI—Plaque Index; PPD—periodontal probing depths; REC—gingival recession; SD—standard deviation; sRANKL—soluble receptor activator of nuclear factor kB ligand; T1DM—type 1 diabetes mellitus; T2DM—type 2 diabetes mellitus; THP-1—human leukemia monocytic cell line; TNF-α—tumor necrosis factor α.

**Table 4:** PICO strategy.

P	Population	Type 1 Diabetic Patients
**I**	intervention/exposure	To evaluate in both gingival crevicular fluid (GCF) and saliva samples the quantification of IL-1β, IL-6, IL-23, IFNγ, IL-1RA and IP-10, as well as IL-10, IL-17, IL-33 and RANK-L, given their described role in the inflammatory dysregulation observed in diabetic patients.
**C**	comparison	Patients without periodontal disease; systemic healthy patients without type 1 diabetes mellitus disease.
**O**	outcome	To analyze the inflammatory biomarkers between type 1 diabetes mellitus and periodontal disease patients.

## Data Availability

The data can be accessed by contacting the corresponding author.
